# Pathway-focused bioassays and transcriptome analysis contribute to a better activity monitoring of complex herbal remedies

**DOI:** 10.1186/1471-2164-14-133

**Published:** 2013-02-27

**Authors:** Angela Klein, Oliver A Wrulich, Marcel Jenny, Peter Gruber, Kathrin Becker, Dietmar Fuchs, Johanna M Gostner, Florian Überall

**Affiliations:** 1 Division of Medical Biochemistry, Innsbruck Medical University, Center for Chemistry and Biomedicine, Innrain 80-82, Innsbruck, Austria; 2 Division of Biological Chemistry, Innsbruck Medical University, Center for Chemistry and Biomedicine, Innrain 80-82, Innsbruck, Austria

**Keywords:** HepG2, Microarray, Multicomponent, Pathway analysis, Polyherbal, qPCR

## Abstract

**Background:**

Transcriptome analysis in combination with pathway-focused bioassays is suggested to be a helpful approach for gaining deeper insights into the complex mechanisms of action of herbal multicomponent preparations in living cells. The polyherbalism based concept of Tibetan and Ayurvedic medicine considers therapeutic efficacy through multi-target effects. A polyherbal Indo-Tibetan preparation, Padma 28, approved by the Swiss drug authorities (Swissmedic Nr. 58436), was applied to a more detailed dissection of mechanism of action in human hepatoma HepG2 cells. Cell-free and cell-based assays were employed to evaluate the antioxidant capacity. Genome-wide expression profiling was done by applying Human Genome U133 Plus 2.0 Affymetrix arrays. Pathway- and network-oriented analysis elucidated the affected biological processes. The results were validated using reporter gene assays and quantitative real-time PCR.

**Results:**

To reveal the direct radical scavenging effects of the ethanolic extract of the Indo-Tibetan polyherbal remedy Padma 28, an *in vitro* oxygen radical absorbance capacity assay (ORAC) was employed, which resulted in a peroxyl-radical scavenging activity of 2006 ± 235 μmol TE/g. Furthermore, the antioxidant capacity of Padma 28 was analysed in living HepG2 cells, by measuring its scavenging potential against radical induced ROS. This formulation showed a considerable antioxidant capacity by significantly reducing ROS levels in a dose-dependent manner.

Integrated transcriptome analysis revealed a major influence on phase I and phase II detoxification and the oxidative stress response. Selected target genes, such as heme oxygenase 1, were validated in qPCR experiments. Network analysis showed 18 interrelated networks involved in important biological functions such as drug and bio-molecule metabolism, molecular transport and cellular communication. Some molecules are part of signaling cascades that are active during development and morphogenesis or are involved in pathological conditions and inflammatory response.

**Conclusions:**

The identified molecular targets and pathways suggest several mechanisms that underlie the biological activity of the preparation. Although extrapolation of these findings to the *in vivo* situation is not possible, the results obtained might be the basis for further investigations and new hypotheses to be tested. This study demonstrates the potential of the combination of focused and unbiased research strategies in the mode of action analysis of multicomponent herbal mixtures.

## Background

Herbal drug combinations have long been used in traditional medicinal concepts, and multicomponent preparations, such as plant-derived pharmaceuticals, botanicals and dietary supplements, are becoming increasingly important in modern medicine and lifestyles. The concept of effect potentiation by synergistic actions has opened new approaches for phytopharmaceutical and nutraceutical product development, and suggests the (re-)utilization of some traditional formulations for therapeutic issues. However, this development is hampered by the difficulties that accompany the assessment of molecular mechanisms and pharmacological actions of multicomponent preparations that contain an enormous chemical diversity of substances [1-3].

One major beneficial effect of many multicomponent preparations is their antioxidant activity. Reactive oxygen species (ROS) are involved in the development and the progression of diseases such as cardiovascular disorders [4], neurodegeneration [5] and atherosclerosis [6]. The capacity of dietary antioxidants to modulate the cellular redox balance is discussed to be favourable in both the treatment and prevention of ROS-related disorders [7,8]. An antioxidant capacity may not only originate from the direct radical scavenging activity of a compound, but also from indirectly induced effects such as interference with the endogenous antioxidant machinery, the transcription induction of detoxifying and cytoprotective enzymes or the increase of immunoprotective mechanisms [9,10]. Several of these actions are mediated by the nuclear-factor-E2-related factor (Nrf)-2 that transactivates the transcription of protective genes upon binding of antioxidant response element (ARE) promoter sequences to maintain homeostasis during oxidative stress [11]. However, an extensive input of antioxidants can also promote adverse responses such as allergies or toxicity-related phenomena [12,13].

Studies into the interference of phytochemicals with cellular signaling pathways and gene expression have revealed many examples of potential biological activities that go far beyond an antioxidant action [14,15]. Further, the use of large-scale data acquisition strategies, such as gene expression analysis, is suggested to be helpful in deciphering complex molecular information flows [16-18]. Currently, DNA microarray technology is the most widely used genome-scale data in pharmacological and clinical research. Transcriptomics allows the simultaneous detection of multiple transcriptional events in a non-biased manner, enabling standards for analysis and interpretation to be continuously developed [19]. Microarray expression data may provide key insights into gene function and interactions within and across metabolic pathways. However, it appears necessary to go beyond simple clustering by integrating data into biological interaction databases [20].

In general, the therapeutic efficacy of botanical multicomponents is suggested to be based on the interference of a multitude of phytochemicals with multiple cellular targets [21,22]. This enormous complexity of interactions represents a challenge for activity monitoring. Often, the active principles of such remedies cannot be identified, as the pharmacological effects are suggested to be generated from the joint actions of several substances. Thus, reductionist and hypothesis-driven research strategies may prove insufficient to deal with such pleiotropic activity profiles. The combination of pathway or molecule focused and large-scale data assessment strategies, such as “omics” technologies, has proved useful in monosubstance and drug combination activity assessment and is also considered helpful for multicomponents [23-25].

Based on these assumptions, we attempted to explore the mode of action of the Indo-Tibetan remedy Padma 28 at the molecular and transcriptional level in human hepatoma HepG2 cells [26]. Padma 28 is a polyherbal preparation containing a variety of secondary plant substances with potential bioactivity, such as essential oils, flavonoids, tanning agents and sesquiterpenes. It contains 20 different powdered plants including natural camphor and calcium sulphate. The formulation has been subjected to extensive *in vitro* and *in vivo* studies and has been approved by the Swiss drug authorities (Swissmedic, Nr. 58436) for symptoms associated with circulatory disorders such as tingling sensation, formication, feeling of heaviness and tension in the legs and arms, numbness of the hands and feet and calf cramps [27]. Studies have been performed where Padma 28 was applied in the treatment of intermittent claudication, a hallmark of peripheral arterial occlusive disease (PAOD) [28,29]. HepG2 cells were used because the liver is the main organ for drug metabolism after the ingestion of compounds and because of their suitability for studying the mechanism of action of drugs, dietary genotoxicants, as well as the cytoprotective and antigenotoxic activities of agents [30,31]. Furthermore, this cell line is useful for the evaluation of pro- and antioxidant agents as they express many related enzymes such as Mn-superoxide dismutase, catalase, glutathione reductase and thioredoxin reductase [32,33]. A HepG2 derived reporter gene cell line, CellSensor® ARE-bla HepG2, has been applied to investigate ARE-mediated transcriptional response.

## Results

### HepG2 cell proliferation

**Figure 1:**
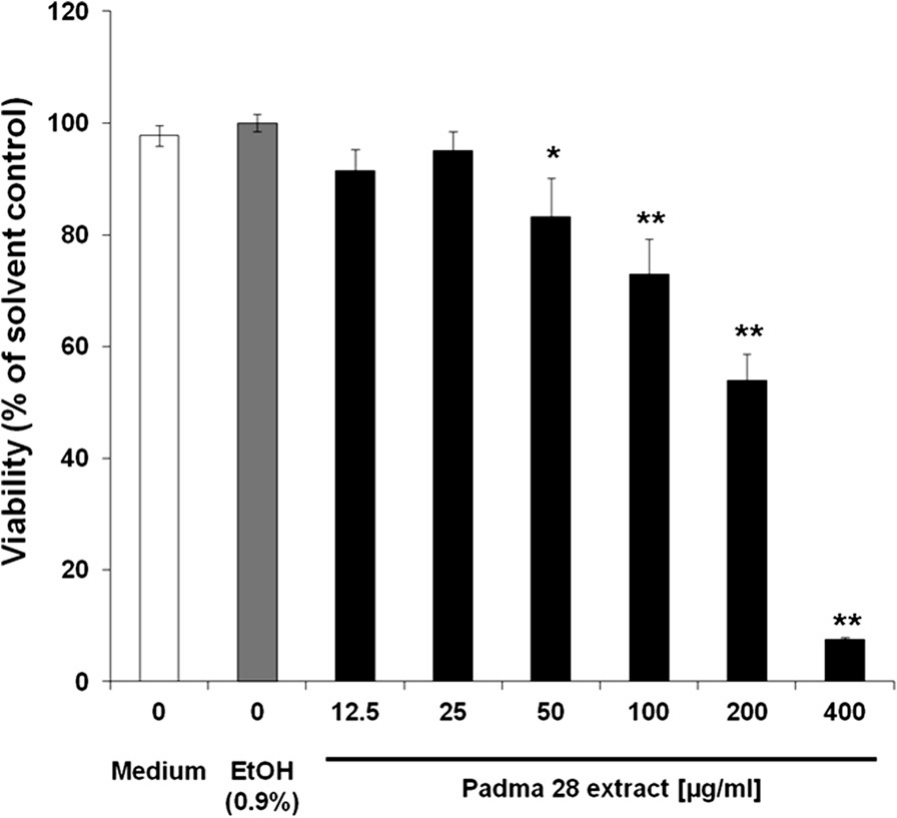
**Effect of Padma 28 on cell viability.** HepG2 cells (2 × 10^4^/well) were seeded into 96-well plates, pre-cultured for 24 h and then treated with solvent (0.9% EtOH) or Padma 28 ethanolic extract (12.5–400 μg/ml) for 72 h. The mean percentages of cell growth relative to solvent-treated cells were plotted against the concentrations of Padma 28. Mean values ± S.E.M. of four independent experiments run in duplicates (*p <0.05; **p <0.005, compared to untreated cells) are shown.

To determine the effect of Padma 28 on cell viability and to define optimal treatment conditions for further cell culture experiments, HepG2 cells were treated with increasing concentrations of the ethanolic extract (12.5–400 μg/ml) and the solvent control (0.9% EtOH, v/v). Cell viability was calculated in relation to the solvent control. As shown in Figure 1, treatment of HepG2 cells with Padma 28 for 72 h dose-dependently decreased the number of viable cells with an IC50-value of 218.4 ± 20.5 μg/ml. 

### Antioxidant capacity

To ensure potent biological activity of the ethanolic extract of Padma 28, its antioxidant capacity was evaluated using the Oxygen Radical Absorbance Capacity (ORAC) assay, which measures the direct capacity of chain-breaking antioxidants based on the hydrogen atom transfer mechanism in a cell-free system. The polyherbal extract showed potent peroxyl-radical scavenging capacity *in vitro*, with an ORAC value of 2006 ± 235 μmol TE/g (data not shown). Accordingly, 1 μg/ml of Padma 28 corresponds to the net protection against peroxyl-radicals produced by about 2 μM or 0.5 μg/ml of the water-soluble vitamin E derivate Trolox.

**Figure 2:**
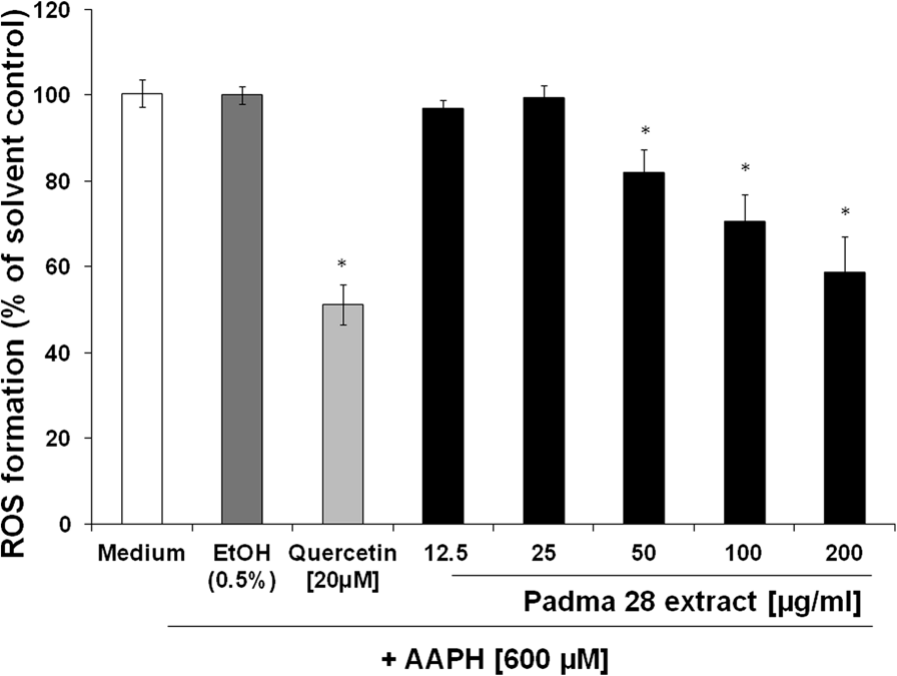
**Measurement of intracellular ROS.** Inhibition of peroxyl-radical (AAPH; 600 μM)-induced formation of ROS in HepG2 cells pretreated with either Quercetin [20 μM], as a positive control, or increasing concentrations of Padma 28 (12.5–200 μg/ml). The mean percentages of DCF fluorescence, as a measure of ROS formation, are shown in relation to the AAPH-treated EtOH solvent control (set to 100%). Mean values ± S.E.M. of three independent experiments run in quadruplicates (*p <0.05, compared to AAPH-treated cells) are shown.

The antioxidant capacity of Padma 28 in living cells was analysed by measurement of peroxyl-radical-induced intracellular ROS-levels in HepG2 cells pretreated with increasing concentrations of ethanolic extract. As shown in Figure 2, exposure of HepG2 cells to Padma 28, at concentrations ranging from 50–200 μg/ml, significantly and dose-dependently diminished 2,2'-azobis(2-amidino-propane) dihydrochloride (AAPH)-induced ROS levels (p<0.05). At a concentration of 200 μg/ml, a significant reduction of AAPH-stimulated ROS levels to 58.7± 8.3% could be observed. In comparison, treatment of cells with 20 μM Quercetin, a flavonoid with potent antioxidant properties, decreased AAPH-induced ROS production to 51.1 ± 2.1%. 

### Gene expression changes

Affymetrix GeneChip Human Genome U133 Plus 2.0 arrays were used to analyse Padma 28 regulated gene expression in HepG2 cells. An experimental approach using only one microarray per treatment (n=1) was applied, in which the cells of three biological replicates were pooled to obtain a “biological average” of stimulated gene expression changes. Particular attention was paid to profound validation of expression data by qPCR. Analysis of differential gene expression was performed by comparison of gene expression patterns of HepG2 cells treated for 18 h with an ethanolic extract of Padma 28, at doses corresponding to the 50% inhibitory concentration (IC50) after 72 h (218 μg/ml), and solvent (0.9% EtOH), respectively. At this concentration, no effect on cell viability was detectable after 24 h of incubation (data not shown).

Microarray analysis revealed a total of 578 genes whose expression was modulated more than two-fold upon extract treatment: 353 genes were up-regulated and 225 down-regulated. Of these, 24 showed a four-fold or higher increase in gene expression, whereas nine genes were down-regulated by a fold change of more than four-fold (Table 1). Selected modulated candidate genes were validated in five independent experiments using qPCR (n=5). As shown in Table 1, nine of the 24 most up-regulated genes and three of the nine most down-regulated genes were significantly positively validated (p <0.05), thus confirming most findings of the microarray analysis. 

**Table 1:** Differentially expressed (>2 log2) genes in HepG2 cells after treatment with Padma 28

		**Microarray (n=1)**		**qPCR (n=5)**	
**Gene**	**Gene title**	**log2 (ratio)**	**Fold**	**Fold**	**P(H1)**
CYP1A1	cytochrome P450, family 1, subfamily A1	8.7	408.4	123.4 ± 31.1	0.001
PTGR1	prostaglandin reductase 1	4.8	27.4		
SLC7A11	solute carrier family 7, member 11	4.2	18.9		
AKR1B10	aldo-keto reductase family 1, member B10	3.9	14.7	13.9 ± 4.9	0.003
ASNS	asparagine synthetase	3.4	10.2	18.8 ± 5.5	0.008
CYP24A1	cytochrome P450, family 24, subfamily A 1	3.1	8.4	5.0 ± 0.9	0.132
PMAIP1	phorbol-12-myristate-13-acetate-induced protein 1	3.0	8.2		
NUPR1	nuclear protein 1	2.6	6.3		
SCHIP1	schwannomin interacting protein 1	2.6	6.2		
GSTA1*	glutathione S-transferase alpha 1	2.6	6.1	4.5 ± 1.3	0.036
FECH	ferrochelatase	2.4	5.2	3.7 ± 0.8	0.018
CD14	CD14 molecule	2.4	5.2		
AKR1C2**	aldo-keto reductase family 1, member C2	2.4	5.1	6.7 ± 1.2	0.007
LOC374443	CLR pseudogene	2.3	5.0		
BCAT1	branched chain aminotransferase 1, cytosolic	2.3	5.0		
KLHDC9	kelch domain containing 9	2.2	4.6		
HELLS	helicase, lymphoid-specific	2.2	4.4		
CARS	cysteinyl-tRNA synthetase	2.1	4.3	3.2 ± 0.6	0.001
AKR1C1**	aldo-keto reductase family 1, member C1	2.1	4.2	6.7 ± 1.2	0.007
ABCC4	ATP-binding cassette, sub-family C, member 4	2.1	4.2		
PIR	pirin (iron-binding nuclear protein)	2.1	4.2		
RAB27A	RAB27A, member RAS oncogene family	2.1	4.2		
HMOX1	haeme oxygenase (decycling) 1	2.1	4.2	5.3 ± 0.8	0.008
GCLM	glutamate-cysteine ligase, modifier subunit	2.1	4.1		
RBM39	RNA binding motif protein 39	-2.8	-7.2	1.1 ± 0.6	0.083
DKK1	dickkopf homolog 1 (Xenopus laevis)	-2.7	-6.7	-24.2 ± 5.9	0.005
SCN1A	sodium channel, voltage-gated, type I, alpha subunit	-2.6	-6.0	-30.3 ± 10	0.002
SLC13A3	solute carrier family 13, member A3	-2.4	-5.2		
MT1M	metallothionein 1M	-2.3	-4.9		
LGR5	leucine-rich G protein-coupled receptor 5	-2.1	-4.4	-8.1 ± 1.9	0.001
SLC26A3	solute carrier family 26, member 3	-2.1	-4.2		
SH3PXD2A	SH3 and PX domains 2A	-2.0	-4.1		
SUCLG1	succinate-CoA ligase, alpha subunit	-2.0	-4.1	0.3 ± 0.6	0.786

*primers detect GSTA1-A3 and A5; **primers detect AKR1C1-C4a.

The P(H1) value calculated for the qPCR experiments indicates the probability that the difference between sample and control group is significant.

### Network and pathway analysis

A total of 41,599 of the 54,675 probe sets could be mapped in the Ingenuity Knowledge Base of the analysis software (IPA) to the corresponding molecule record [34]. Using a cut-off value of 1.0 (log2 ratio = two-fold up- or down-regulated), IPA revealed 324 genes eligible for network generation and 318 genes eligible for pathway analysis. In the network analysis, differentially regulated genes are inter-connected using information of known interactions and associations between genes or proteins extracted from literature and databank findings.

Network analysis of >two-fold differentially expressed genes in HepG2 cells after treatment with Padma 28 [218 μg/ml] for 18 h, revealed 25 networks, of which 18 displayed various degrees of interrelationship. A list of networks with a high score of >20 and the corresponding differentially expressed molecules identified is provided as supplementary file (Additional file 1: Table S1). These genes are associated with signaling pathways essential for the metabolic processing of drugs, amino acids, lipids, small molecules, vitamins and minerals. Besides affecting general mechanisms such as molecular transport, cell-to-cell signaling and interaction, some molecules are part of signaling cascades that are active during development and morphogenesis or play a role in pathological conditions such as genetic disorders, cancer and connective tissue disorders.

**Figure 3:**
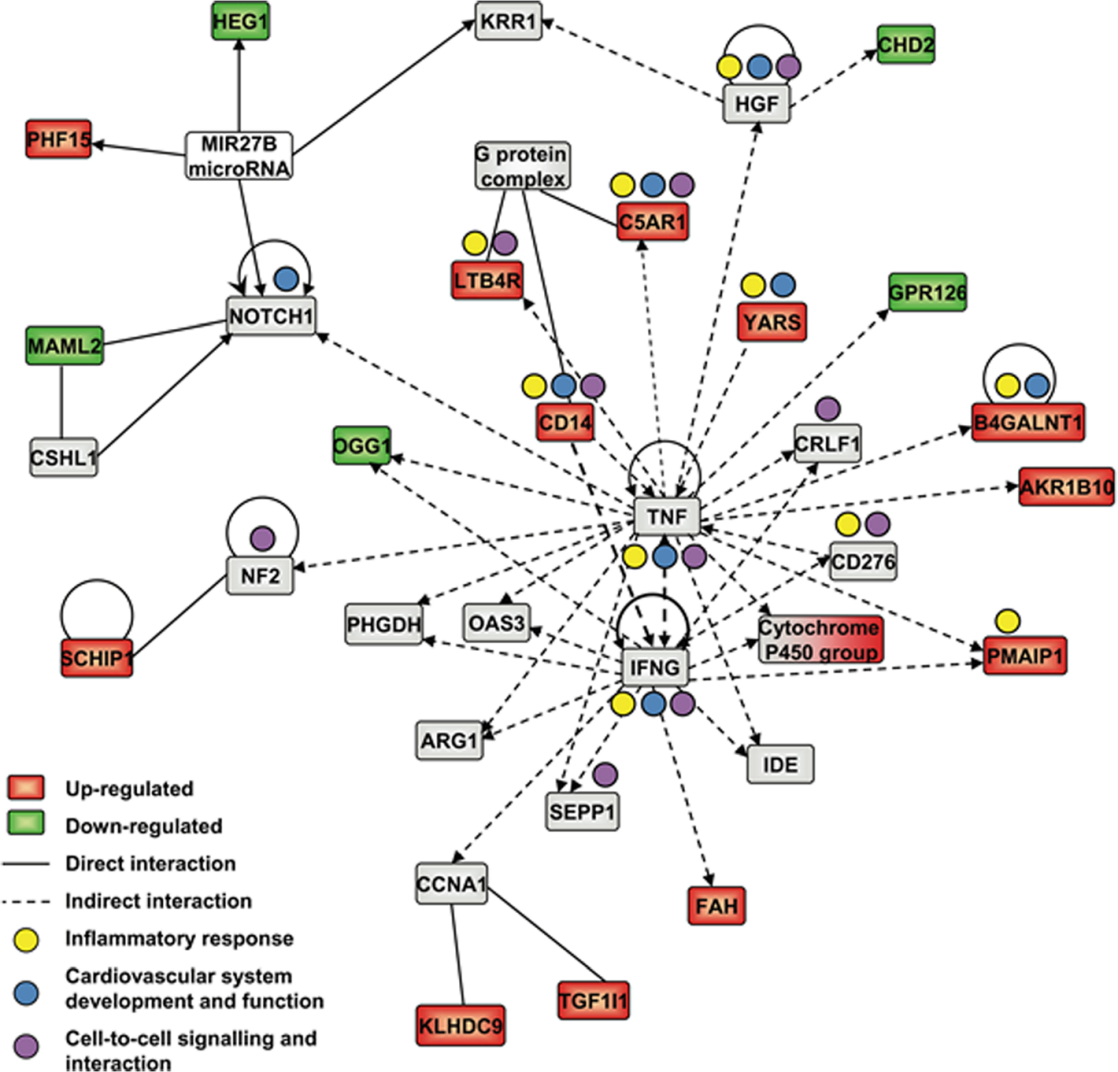
**Interactions of network 7.** Graphical presentation of network 7, obtained by IPA network analysis of microarray data of differential gene expression in HepG2 cells after exposure to 218 μg/ml Padma 28 ethanolic extract for 18 h.

**Figure 4:**
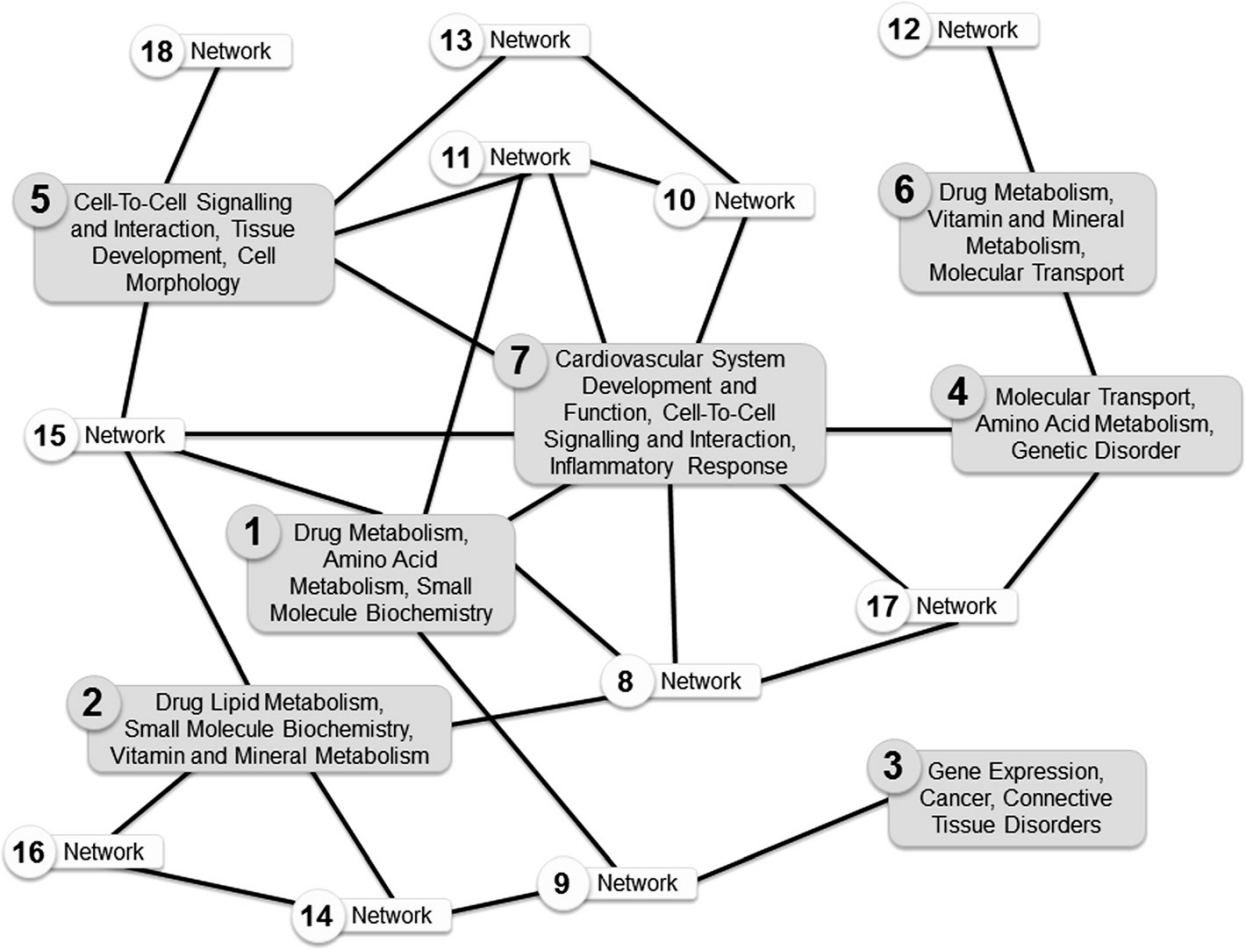
**Cellular processes and functions.** Overview on the network connections extracted from the findings of altered gene expression in HepG2 cells following Padma 28 treatment. Ingenuity network analysis identified 25 networks, of which 18 displayed various degrees of interrelationship such as molecules influencing other components within a network or molecules participating in more than one biological process, thus appearing in more than one network. The top seven networks with a score >20 are highlighted and labelled. Both up- and down-regulated identifiers were included in the analysis.

Interestingly, network analysis revealed that Padma 28 also modulates networks associated with cardiovascular system development and function as well as with inflammatory responses (network 7, Figure 3 and Additional file 1: Table S1), which appear to be central within the overlapping networks identified. Figure 4 shows the relationships of 18 overlapping networks, of which the top seven (with a score >20; Table 2), are highlighted in grey. 

Furthermore, 21 canonical pathways were identified from the IPA library, which appeared with significance (p<0.05) in the dataset. The top six canonical pathways (p-value in the range of 3.8 × 10^-5^ to 4.7 × 10^-3^) are involved in the metabolism of xenobiotics by cytochrome P450 (CYP), glutathione metabolism, aminoacyl-tRNA biosynthesis, lipopolysaccharide (LPS)/interleukin-1 (IL-1)-mediated inhibition of retinoid X receptor (RXR) function, Nrf2-mediated oxidative stress response and aryl-hydrocarbon receptor (AhR) signaling (Table 2). qPCR analysis positively validated the differential expression of at least two genes in each pathway in five independent experiments (p<0.05). 

**Table 2:** Top canonical pathways and corresponding genes modulated by Padma 28 in HepG2 cells

		**Microarray (n=1)**		**RT-PCR (n=5)**	
**Pathway/Gene**	**Gene title**	**log2 (ratio)**	**Fold**	**Fold**	**P(H1)**
**Metabolism of xenobiotics by cytochrome P450** (*p*-value: 3.82E-05; ratio: 10/178)					
CYP1A1	cytochrome P450, family 1, subfamily A1	8.7	408.4	123.4 ± 31.1	0.001
PTGR1	prostaglandin reductase 1	4.8	27.9		
GSTA1	glutathione S-transferase alpha 1	2.6	6.1	4.5 ± 1.3	0.036
AKR1C2	aldo-keto reductase family 1, member C2	2.4	5.3	6.7 ± 1.2	0.007
AKR1C1	aldo-keto reductase family 1, member C1	2.1	4.3	6.7 ± 1.2	0.007
CYP4F11	cytochrome P450, family 4, subfamily F 11	1.7	3.2		
MGST1	microsomal glutathione S-transferase 1	1.6	3.0		
ALDH1L1	aldehyde dehydrogenase 1 family, member L1	1.6	3.0	4.1 ± 1.0	0.003
EPHX1	epoxide hydrolase 1, microsomal	1.3	2.5		
CYP2S1	cytochrome P450, family 2, subfamily S1	1.2	2.2	5.1 ± 1.5	0.018
**Glutathione metabolism** (*p*-value: 4,01E-04; ratio 7/92)					
GSTA1	glutathione S-transferase alpha 1	2.6	6.1	4.5 ± 1.3	0.036
GCLM	glutamate-cysteine ligase, modifier subunit	2.1	4.1		
GCLC	glutamate-cysteine ligase, catalytic subunit	1.7	3.3	5.8 ± 0.9	0.009
MGST1	microsomal glutathione S-transferase 1	1.6	3.0		
GPX2	glutathione peroxidase 2	1.2	2.3	0.3 ± 0.9	0.881
RAB15	RAB15, member RAS onocogene family	1.1	2.1		
IDH3A	isocitrate dehydrogenase 3 (NAD+) alpha	1.1	2.1		
**Aminoacyl-tRNA biosynthesis** (*p*-value: 4.24E-04; ratio 6/83)					
CARS	cysteinyl-tRNA synthetase	2.1	4.3	3.2 ± 0.6	0.001
YARS	tyrosyl-tRNA synthetase	1.3	2.5		
WARS	tryptophanyl-tRNA synthetase	1.2	2.4	3.8 ± 1.0	0.020
GARS	glycyl-tRNA synthetase	1.2	2.3		
AARS	alanyl-tRNA synthetase	1.2	2.3		
MARS	methionyl-tRNA synthetase	1.1	2.2		
**LPS/IL-1 mediated inhibition of RXR function** (*p*-value: 1.82E-03; ratio 12/205)					
GSTA1	glutathione S-transferase alpha 1	2.6	6.1	4.5 ± 1.3	0.036
CD14	CD14 molecule	2.4	5.3		
ABCC4	ATP-binding cassette, sub-family C, member 4	2.1	4.3		
ABCC3	ATP-binding cassette, sub-family C, member 3	1.8	3.5		
MGST1	microsomal glutathione S-transferase 1	1.6	3.0		
ALDH1L1	aldehyde dehydrogenase 1 family, member L1	1.6	3.0	4.1 ± 1.0	0.003
NR1H3	nuclear receptor subfamily 1, group H, member 3	1.5	2.8		
ALDH1A1	aldehyde dehydrogenase 1 family, member A1	1.3	2.5		
SLC35A2	solute carrier family 35, member A2	1.1	2.1		
SULT1E1	sulfotransferase family 1E, member 1	-1.3	-2.5		
SLCO1B3	solute carrier organic anion transporter 1B3	-1.3	-2.5		
FMO5	flavin containing monooxygenase 5	-1.9	-3.7		
**Nrf2-mediated oxidative stress response **(*p*-value: 2.92E-03; ratio 11/181)					
GSTA1	glutathione S-transferase alpha 1	2.6	6.1	4.5 ± 1.3	0.036
HMOX1	heme oxygenase (decycling) 1	2.1	4.3	5.3 ± 0.8	0.008
GCLM	glutamate-cysteine ligase, modifier subunit	2.1	4.3		
GCLC	glutamate-cysteine ligase, catalytic subunit	1.7	3.3	5.8 ± 0.9	0.009
NQO1	NAD(P)H dehydrogenase, quinone 1	1.7	3.2		
MGST1	microsomal glutathione S-transferase 1	1.6	3.0		
EPHX1	epoxide hydrolase 1, microsomal (xenobiotic)	1.3	2.5		
FTH1	ferritin, heavy polypeptide 1	1.3	2.5		
TXNRD1	thioredoxin reductase 1	1.3	2.5		
GPX2	glutathione peroxidase 2 (gastrointestinal)	1.2	2.3	0.3 ± 0.9	0.881
SLC35A2	solute carrier family 35 , member A2	1.1	2.1		
**Aryl Hydrocarbon Receptor Signaling** (*p*-value: 4.65E-03; ratio 9/150)					
CYP1A1	cytochrome P450, family 1, subfamily A1	8.7	408.4	123.4 ± 31.1	0.001
GSTA1	glutathione S-transferase alpha 1	2.6	6.1	4.5 ± 1.3	0.036
NQO1	NAD(P)H dehydrogenase, quinone 1	1.7	3.2		
FAS	Fas (TNF receptor superfamily, member 6)	1.7	3.2		
MGST1	microsomal glutathione S-transferase 1	1.6	3.0		
ALDH1L1	aldehyde dehydrogenase 1 family, member L1	1.6	3.0	4.1 ± 1.0	0.003
ALDH1A1	aldehyde dehydrogenase 1 family, member A1	1.3	2.5		
SLC35A2	solute carrier family 35, member A2	1.1	2.1		
NCOA2	nuclear receptor coactivator 2	-1.1	-2.1		

The p-value is a measure of the probability of the association of a specific pathway and the dataset. The ratio indicates the number of molecules in a given pathway from the input dataset that meet the cut-off criteria (fold change of 2) divided by the number of molecules that make up that pathway in the database.

### Effect of treatment on Nrf2-mediated oxidative stress response

**Figure 5:**
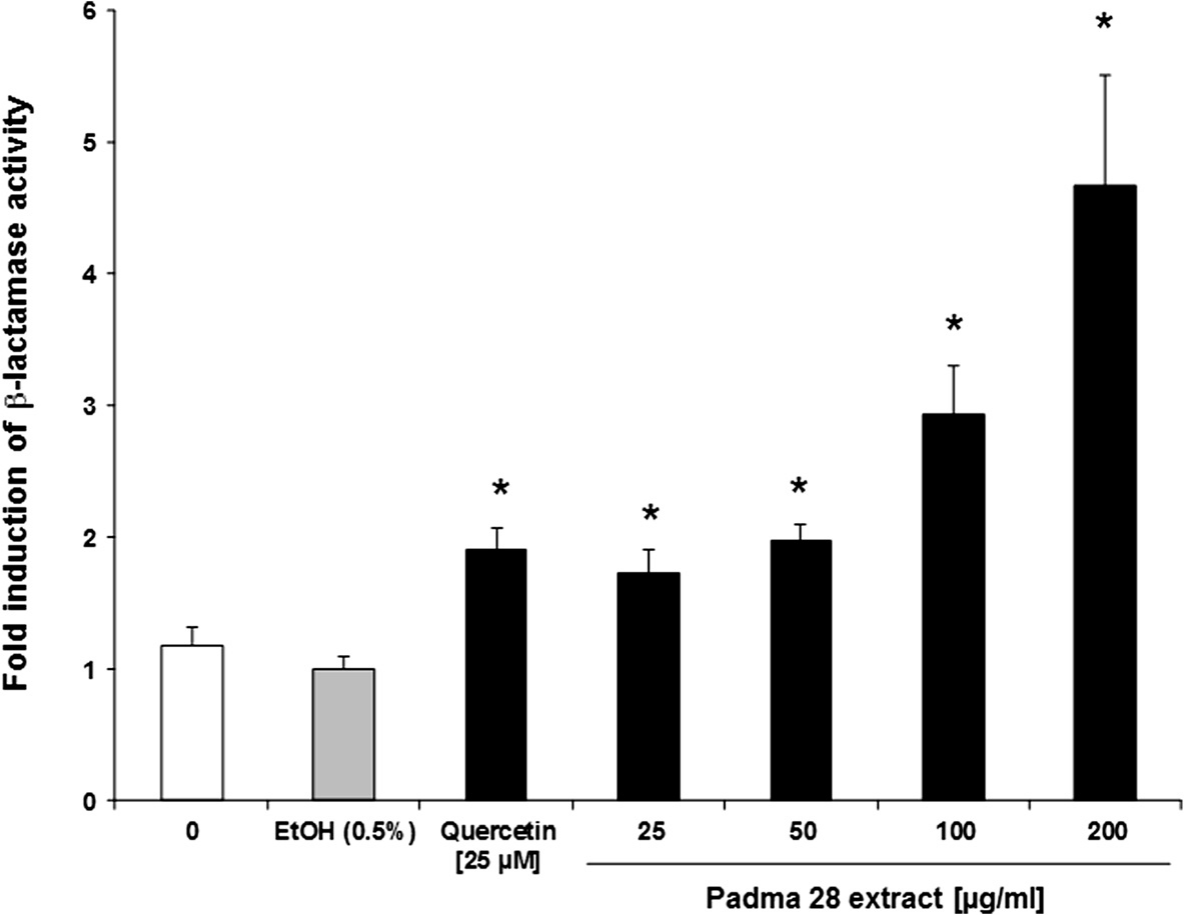
**Activation of the antioxidant response element (ARE)-driven β-lactamase reporter gene expression.** CellSensor® ARE-bla HepG2 cells were treated with Quercetin [25 μM], as a positive control, and increasing concentrations of Padma 28 (25–200 μg/ml) for 18 h. The mean fold induction of β-lactamase activity, as a measure of ARE-mediated transcriptional activation, is shown relative to the solvent control (set to 1). Mean values ± S.E.M. of four independent experiments run in quadruplicates (*p<0.05, compared to the solvent control) are shown.

Based on the observation that Padma 28 up-regulates various genes of the Nrf2-mediated oxidative stress response in the microarray analysis and the fact that Nrf2 is a transcriptional activator of ARE-mediated gene expression, we used the CellSensor® ARE-bla HepG2 cell system to verify Padma 28-induced transcriptional activation of ARE-driven reporter gene expression. As shown in Figure 5, treatment of HepG2 cells with Padma 28 for 18 h dose-dependently stimulated ARE-driven β-lactamase expression. Within a concentration range of 25–200 μg/ml, Padma 28 induced ARE-mediated transcriptional activity 1.7 ± 0.2 to 4.7 ± 0.8-fold. Quercetin at 25 μM showed nearly the same transactivation potency (1.9 ± 0.2-fold induction) as PADMA 28 at doses of 25 μg/ml. 

### Effect on heme oxygenase 1 gene (HMOX) and protein (HO-1) expression

**Figure 6:**
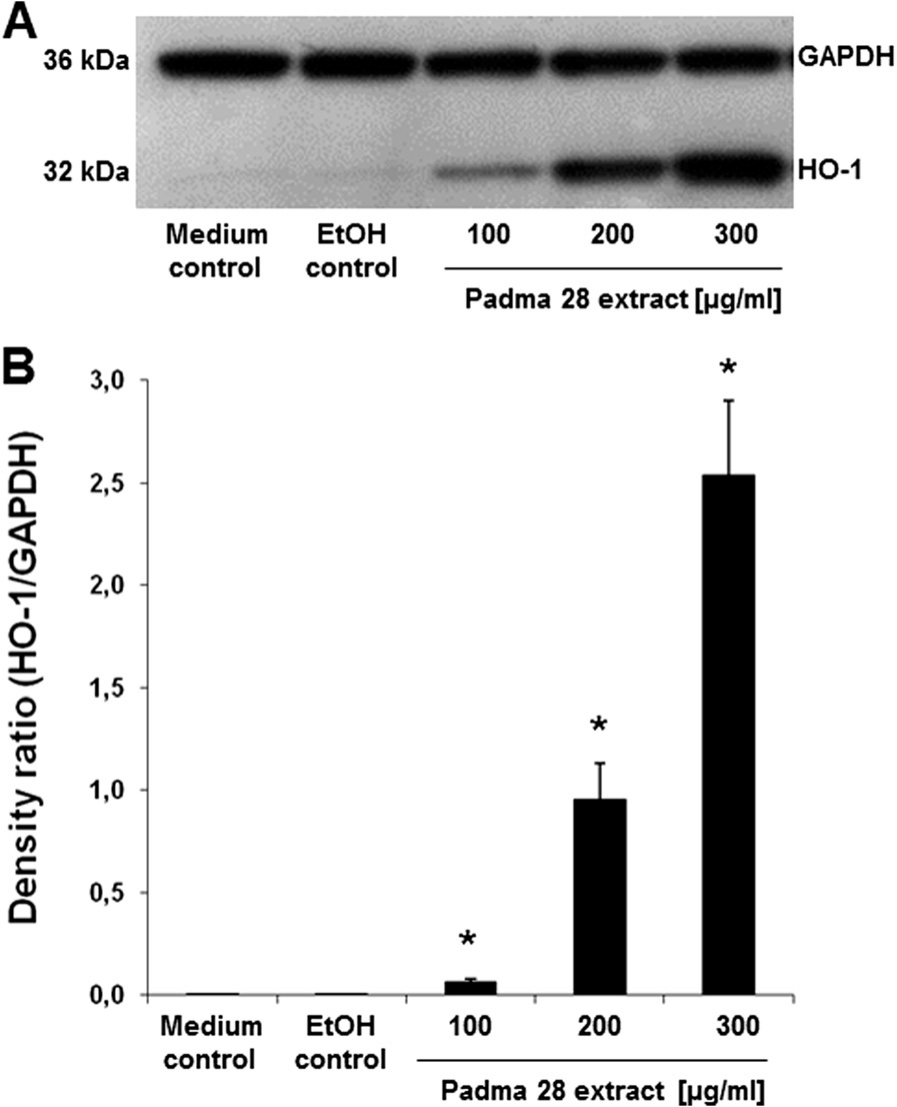
**Heme oxygenase-1 (HO-1) protein expression.** Stimulation of heme oxygenase-1 (HO-1) expression after treatment of HepG2 cells with increasing concentrations of Padma 28 (100–300 μg/ml) for 18 h. (**A**) A representative western blot of HO-1 protein expression is shown. (**B**) Densitometric analysis of HO-1/GAPDH expression. Mean values ± S.E.M. of three independent experiments (*p<0.05, compared to the solvent control) are shown.

Based on the finding that inducible HMOX was up-regulated in the microarray experiment (4.3-fold), as validated by qPCR (5.3 ± 0.8-fold), western blot analysis was performed under the same culture conditions to confirm the up-regulation of HO-1 at the protein level. As shown in Figure 6, expression of HO-1 was dose-dependently induced after treatment of HepG2 cells with Padma 28 ethanolic extract for 18 h, whereas HO-1 protein was undetectable in untreated and solvent-treated cells. 

## Discussion

In traditional medical systems, combinations of herbs and natural materials are often used in the treatment of multifactorial diseases [35]. However, evaluation of the molecular mechanisms modulated by complex multicomponent preparation in living cells is often limited because of the chemical diversity and multiple effects on a variety of molecular targets. Functional redundancy and regulatory loops add further complexity to the interaction networks responsible for biological actions [36,37]. Re-establishment of a healthy state following disease development requires interventions on multiple targets at different levels [38-40].

Here, we employed the effects of the well-studied Indo-Tibetan polyherbal remedy Padma 28 on HepG2 cells using a microarray approach to gain deeper insights into multi-target effects of polyherbal preparations.

We used the ORAC method to reveal a peroxyl-radical scavenging activity of 2006 ± 235 μmol TE/g, which is much higher than that of other herbal preparations. Liao et al. reported an ORAC range of 40–940 μmol TE/g for 35 herbs used in traditional Chinese medicine, with a further eight showing antioxidant capacities of 1000–1500 μmol TE/g and only two approaching the level of Padma 28 [41]. Of note, the antioxidant capacity of herbal preparations may depend not only from the plant species used, but also from other factors e.g. the extract processing procedure or the storage under non oxidizing conditions. Also, in cultivated HepG2 cells, Padma 28 ethanolic extract showed a considerable antioxidant capacity by dose-dependently decreasing peroxyl-radical-induced intracellular ROS levels. These results are consistent with previous reports about the concentration dependent antioxidant and pro-oxidant ability of Padma 28 [42,43]. Beside peroxyl radical, other ROS such as superoxide, hydrogen peroxide, hydroxyl and alkoxyl radicals, as well as reactive nitrogen species (RNS) are produced under (patho-)physiological conditions and may be neutralized by antioxidant compounds [44,45].

To investigate genome-wide transcriptional alterations in treated HepG2 cells, we used a concentration of the extract equal to the IC50 value of growth after 72 h (218 μg/ml), but limited the incubation period to 18 h, where cell viability was not yet affected (data not shown). At this concentration, AAPH-induced ROS level in HepG2 cells was significantly decreased. A total of 578 genes were more than two-fold differentially regulated following Padma 28 treatment compared to the solvent control. However, the extent of regulation was mostly moderate and only 24 genes showed a four-fold or higher increase in gene expression, whereas nine were down-regulated at least four-fold. Notably, despite the weakness of regulation, the gene expression change of 12 selected transcripts was confirmed by qPCR in five independent experiments, implicating the robustness of the experimental design. According to the biological response fingerprinting (BioReF) approach of Rong et al. [46], this set of validated transcripts may be used as a signature pattern, which may be specific for the biological response of Padma 28 in HepG2 cells. Thus, qPCR analysis of marker genes might serve as a sensitive and cost-effective method for quality assessment and batch-to-batch comparison of polyherbal formulations. However, these results should be confirmed by further experiments using different incubation times, treatment concentrations and other cell models.

One of the major findings of this microarray analysis was the differential expression of phase I and II metabolic enzymes upon treatment of HepG2 cells with Padma 28. The CYP superfamily plays an essential role in detoxification phase I metabolism reactions of a wide range of xenobiotics including therapeutic agents, food additives, cruciferous vegetables and herbal components [47-50]. The majority of CYP-mediated drug biotransformation reactions are catalysed by CYP1A2, 2C9, 2C19, 2D6 and 3A4, and, to a limited extent, CYP2C8, 2B6 and 3A5 [47,51]. Other CYP isoenzymes such as CYP1A1, 1B1, 2A6, and 2E1 are modulated by only a few, if any, therapeutics [51].

In the present study, the isoenzyme CYP1A1 was differentially expressed to the greatest extent. It is of a particular interest because of its implication in the metabolic activation of some exogenous pro-carcinogens or endogenous molecules [52]. Interestingly, CYP1A1 also exerts important functions in the detoxification of environmental carcinogens and the metabolic conversion of dietary compounds into substances with cancer preventive properties [53,54]. As many phytochemicals are known to interact with CYP1A1 and AhR signaling, a deeper examination of the regulation of CYP1A1 by herbal components is of pharmacological relevance [55]. In general, CYP1A1 expression is mediated by the aryl hydrocarbon receptor (AhR) transcription factor. Additionally, AhR signaling is involved in many cellular processes such as cell growth, apoptosis, hypoxia signaling, cell adhesion and cell matrix metabolism [56-58]. AhR induces the expression of several phase II xenobiotic metabolizing enzymes, such as glutathione S-transferase alpha 1 (GSTA1), NAD(P)H: quinone oxidoreductase 1 (NQO1), microsomal glutathione S-transferase 1 (MGST1) or aldehyde dehydrogenase 1 family member L1 (ALDH1L1). Interestingly, in the present study, several AhR regulated enzymes were up-regulated by Padma 28 treatment in HepG2 cells (Table 2).

The effect of Padma 28 on cell growth and apoptosis has been illustrated by the >six-fold increase of nuclear protein 1 (NUPR1), which responds to proapoptotic stimuli and promotes cellular growth to counteract diverse injuries [59]. In addition, up-regulation of NQO1 by Padma 28 indicates an involvement of apoptosis and hypoxia signalling, since NQO1 is able to stabilize the p53 tumour suppressor protein, especially under oxidative stress conditions [60].

As expected from a pleiotropic acting antioxidant remedy, Nrf2-regulated stress response genes were also differentially expressed following Padma 28 treatment (Table 2). Nrf2 is a transcriptional activator for the ARE-mediated induction of antioxidant and phase II-detoxifying genes such as HMOX or glutathione S-transferase (GST), which protect cells and tissues from oxidative damage [11]. To validate this result, we showed that ARE-promoter driven β-lactamase expression was dose-dependently induced in a reporter cell line transactivation assay (Figure 5). We also observed a significant up-regulation of further antioxidant and detoxification genes such as HMOX, glutamate-cysteine ligase modifier (GCLM) and catalytic (GCLC) subunits, aldo-keto reductase family 1, member C1 (AKR1C1) and member C2 (AKR1C2) in the microarray analysis (Table 2). The induction of heme oxygenase expression upon treatment of HepG2 cells with Padma 28 could be confirmed at both the transcript (Table 1) and protein level (HO-1, Figure 6). The expression of heme oxygenase-1 is associated with therapeutic benefits in a number of pathologic conditions such as atherosclerotic vascular disease and inflammation [61]. A number of natural and synthetic molecules have been reported to induce HO-1 as additive mechanism responsible for their therapeutic effects [62].

The network visualization of the interaction of differentially expressed genes provides a link between transcriptional changes and their functional consequences in biological processes. We observed interference of the herbal preparation with molecules relevant to cardiovascular system development and function, cell-to-cell signaling and interaction and inflammatory responses (Figure 3 and Additional file 1: Table S1, network 7) within a centrally arranged subnetwork. Within this subnetwork, tumor necrosis factor (TNF) and interferon gamma (IFNG) have a central position. TNF is a principal mediator of the acute inflammatory response and coordinates the proliferation and protective functions of pathogen-reactive cells [63]. TNF is engaged in the activation of various pathways under both pathological and physiological conditions, and shows a remarkable functional duality, thus being also responsible for many of the systemic complications of severe infections [64]. The pro-inflammatory cytokine interferon gamma (IFNγ) plays a central role in the cellular immune response, as it induces several immuno-regulatory pathways and cellular response signals e.g. via the Janus activated kinase (JAK)-signal transducer and activator of transcription (STAT) pathway [65,66]. Beside anti-microbial and inflammatory responses, IFNγ also interferes with growth suppression, cell death, tumor immunity and autoimmunity [66].

The strong interconnection of network 7 with other subnetworks might indicate that the represented interactions are involved in the regulation of some important cell behavior, as the removal of this part would affect the whole interaction network [38]. However, it should be kept in mind that transcriptional signatures are time and dosage dependent. Therefore, these results should be further validated using additional experimental approaches in order to design a more comprehensive activity model of Padma 28 that reflects also low-dose and long term effects. The application of “omics” technologies in multicomponent activity assessment is suggested to be most useful when applied in an iterative strategy in combination with focused bioassay [23]. Moreover, the interaction databases used for network generation might influence the result. Thus, our experimental findings must be interpreted with caution and a broader experimental design is necessary to use such data in the context of a comprehensive risk-benefit assessment.

## Conclusions

In the present study, we show that the Indo-Tibetan polyherbal remedy Padma 28 displays both direct and indirect antioxidant capacities and exerts a variety of actions including the transcriptional activation of cytoprotective genes. The identification of a number of potential key molecules and signaling cascades that are differentially regulated upon cell treatment *in vitro*, supports the hypothesis for a pleiotropic mode of action. The results agree with several previous studies that described antioxidant, anti-inflammatory and anti-atherosclerotic properties using different experimental approaches.

The combination of molecular and pathway-based bioassays with unbiased transcriptome analysis has proved to be a valuable strategy in multicomponent activity assessment. Nevertheless, the selection of an appropriate experimental design, the cellular model system and the verification of findings in an iterative manner is necessary to generate valuable results.

## Methods

### Preparation of polyherbal extracts

The Indo-Tibetan polyherbal preparation Padma 28 (*Swissmedic Nr.* 58436) was provided by PADMA Inc. (Schwerzenbach, Switzerland) and contains twenty individual herbs (Aegle sepiar fructus, Amomi fructus, Aquilegiae vulgaris herba, Calendulae flos cum calyce, Cardamomi fructus, Caryophylli flos, Costi amari radix, Kaempferiae galangae rhizome, Lactucae sativae folium, Lichen islandicus, Liquiritiae radix, Meliae tousend fructus, Myrobalani fructus sine semine, Plantaginis lanceolatae folium, Polygoni avicularis herba, Potentillae aureae herba, Santali rubri lignum, Sidae cordifoliae herba, Valerianae radix and Aconiti tuber as well as D-Camphora and Calcii sulfas hemihydricus). The raw materials cultivation fulfills the basic requirements of the European Medicines Agency-Good Agricultural Procedure (EMA-GACP) standards. All raw materials are processed according to Good Manufacturer Practice (GMP) guidelines. Pharmaceutical analysis of 1 g of the preparation was performed by Phytolab (Vestenbergsgreuth, Germany, http://www.phytolab.com/de) and revealed the composition of 2.1% essential oils, 0.1% flavonoids, 2.9% tanning agents, 0.006% sesquiterpenes, 2.34% ortho-dihydroxycinnamic acid, 0.012% imperatorin and 0.37% glycyrrhizic acid.

5 g of Padma 28 powder was extracted using 25 ml of 70% ethanol (v/v) at room temperature for 24 h. After centrifugation (4.000 × g for 30 min), the supernatant was sterile filtered and stored light protected at room temperature for analysis. The final concentration of the extract was determined by vacuum evaporation using a rotary evaporator at 40°C and weighting the remaining mass of the extract, which revealed a yield of 33.1 ± 2.2% of the starting material. The final concentration of the extract used for analysis was indicated as mg dry weight/ml of solvent.

### Oxygen Radical Absorbance Capacity (ORAC) assay

The ORAC assay was applied to determine the capacity to scavenge peroxyl-radicals after extraction with 70% ethanol and was performed as described previously [67,68]. The final ORAC values were expressed as μmol Trolox equivalents per gram of dried powder (μmol TE/g).

### Cell culture

The human hepatocellular carcinoma cell line HepG2 (DSMZ, Germany) was cultured in Dulbecco’s Modified Eagle’s Medium (DMEM, Gibco, Germany) supplemented with 10% (v/v) dialyzed fetal bovine serum (FBS, Gibco, Germany) at 37°C in a humidified atmosphere containing 5% CO2.

For cultivation of the CellSensor® ARE-bla HepG2 cell line (Invitrogen, Austria) the medium was additionally supplemented with 0.1 mM non-essential amino acids, 100 U/ml penicillin, 100 μg/ml streptomycin and 5 μg/ml blasticidin (all Invitrogen, Austria). This reporter gene cell line was used to investigate the transcriptional activation of ARE-mediated gene expression upon Padma 28 treatment.

During the experiments, the cultures were maintained in antibiotic-free medium.

### Cell viability assay

To measure viability of HepG2 cells the CellTiter-Blue™Cell Viability Assay (Promega, Germany) was used, which provides a fluorometric method using the indicator dye resazurin to estimate the number of viable cells. HepG2 cells (2 × 10^4^/well) were seeded into 96-well plates, cultured for 24 h and then either left untreated or treated with solvent (0.9% EtOH, v/v) or increasing doses of extract (12.5 - 400 μg/ml) for 72 h. Thereafter, 10% (v/v) CellTiter-Blue™ reagent was added. After 2 h of incubation the fluorescence at 544/590 nm was determined using a Fluoroskan Ascent FL plate-reader (Thermo Labsystems, USA). The half maximal (50% inhibitory) concentration (IC50) was calculated using the original concept of Chou and Talalay by using the CalcuSyn software (Biosoft, UK) [69].

### Measurement of intracellular ROS

Relative changes of intracellular ROS levels in HepG2 cells were monitored by using the fluorescent probe 2’,7’-dichlorofluorescin diacetate (DCFH-DA) as a substrate and 20 μM Quercetin (Sigma-Aldrich, Austria) as a positive control, as described previously [70]. DCFH-DA diffuses through cell membranes and is hydrolyzed by intracellular esterases to non-fluorescent 2',7'-dichlorofluorescin (DCFH), which is subsequently trapped within the cell. In the presence of ROS, DCFH is rapidly oxidized to highly fluorescent 2'-7'- dichlorofluorescein (DCF). The intensity is proportional to the amount of intracellular ROS [71,72].

### RNA preparation

After 24 h of cultivation (5×10^6^ HepG2 cells/ T-75 flask), triplicates of sub-confluent cells were incubated with solvent (0.9% EtOH v/v) or the extract at a concentration corresponding to the IC50 value determined after 72 h (218 μg/ml). 18 h after treatment, total RNA was isolated from pooled cells of extract- and solvent-treated cells using RNeasy Mini Kit (Qiagen, Germany). A DNase (Qiagen, Germany) digest step was added to the protocol. The isolated RNA was quantified at 260/280nm and the quality of total RNA was controlled by the ratio of 18S/28S ribosomal band intensities in an ethidium bromide-containing 1% agarose gel after electrophoresis.

### Gene expression analysis

Gene expression analysis was performed using Affymetrix Human Genome U133 Plus 2.0 Arrays (Affymetrix, Santa Clara, CA). cRNA synthesis, labelling as well as chip hybritisation and image scanning was performed according to the Affymetrix protocol by ImaGene (Germany). All data preprocessing was done in R version 2.8.1 (R Development Core Team 2008). In order to calculate absolute expression values, the BioC GC Robust Multi-array Average (GCRMA) package R version 2.14.1 was used for background correction, normalization, and summarization [73,74]. Background correction is based on a model, which includes GC content information from the probe sequences. As a normalization algorithm, GCRMA uses quantile normalization [75]. The summarization step is a robust multichip model using median polish. Relative log2 ratios were calculated by subtracting the preprocessed absolute expression values of the control group from the treatment group. Unspecific filtering was applied to remove uninformative probe-sets without the use of a priori phenotypic data, to reduce the number of probe sets that have been used in subsequent data mining. The probe sets were ranked according to their relative expression values. A log2 ratio threshold of higher/lower than 1 (fold change > 2) and -1 (fold change < -2), was used. Microarray data are deposited at Gene Expression Omnibus (GSE40580).

### Pathway and network analysis

Ingenuity® Pathway Analysis (IPA, http://www.ingenuity.com, Ingenuity® Systems, USA) was used to inter-connect differential expressed genes in a context specific manner within signaling and metabolic pathways, molecular networks and biological processes that are most significantly perturbed. A cut off value of 1.0 (log2 ratio) was set as a threshold for up- and down-regulation. IPA-Core analysis integrates experimental data with its knowledge base containing a repository of molecular interactions and functional annotations extracted from selected, manually curated, literature sources and databases. IPA version 8.6 (content version 3002) has been used with following filter settings: (species = Uncategorized (e.g. chemicals) OR Human) AND (data sources = Additional interactions OR Ingenuity Expert Findings OR MicroRNA-mRNA interactions OR Protein-protein interactions).

The significance of the association between the data set and the canonical pathways identified was measured by Fisher’s exact test. This was followed by Benjamini-Hochberg (BH) multiple testing correction to calculate a p-value determining the probability that the association between the genes in the data set and the canonical pathway can be accounted for by chance only. The generated networks were ranked by a score based on the inverse log of a p-value generated using the same Fisher’s exact test as above. This considers the number of genes that participated in a given network relative to the total number of genes in the global molecular network stored in the Ingenuity pathway knowledge base. A score of three indicates that there is a 1/1,000 chance that the focus genes are in a network due to random chance. Therefore, scores of three or higher have a 99.9% confidence level of not being generated by random chance alone.

### ARE-GeneBLAzer β-lactamase reporter gene assay

The CellSensor® ARE-bla HepG2 (Invitrogen, Austria) cell line contains a reporter system, in which the expression of the bacterial β-lactamase gene is controlled by a cis-acting ARE response element, which can be induced through the corresponding endogenous transcription factor Nrf2. 7.5 × 10^4^ ARE-bla HepG2 cells/ well were plated into a 96-well plate. 7 h after seeding, the cells were either left untreated or treated with quercetin [25 μM] as a positive control in the presence of 0.5% DMSO (v/v) or extract (25 - 200 μg/ml) in the presence of 0.5% EtOH (v/v). 18 h after treatment, cells were loaded with LiveBLAzer™-FRET B/G substrate CCF4-AM (Invitrogen, Austria) for 2 h, according to the manufacturer’s protocol. β-lactamase expression was determined by enzyme-mediated cleavage of the fluorescence resonance transfer (FRET) substrate [76]. Fluorescence emissions (414/460 nm and 414/538 nm) were measured with a Fluoroskan Ascent FL plate-reader (Thermo Labsystems, USA). The response ratios were calculated as the mean fold induction of beta-lactamase activity relative to the solvent control (set to 1).

### Quantitative real-time polymerase chain reaction (qPCR)

Single-stranded cDNA was synthesized from 1 μg total RNA using random primers and 200 U SuperScript II Reverse Transcriptase (Invitrogen, Austria) according to the manufacturer’s instructions. qPCR analysis was performed in a total volume of 20 μl, using 20 ng cDNA, 200 nM specific primers and SensiMixPlus SYBR (Bioline, Austria) on the Mx3005P cycler (Stratagene, Netherlands) under the following conditions: 95°C 600 sec; 40 cycles: 95°C 15 sec, 60°C 15 sec, 72°C 20 sec (fluorescence acquisition). All cDNAs were analyzed in triplicates from five independent experiments. For each primer pair (see Additional file 1: Table S2) the PCR amplicon length was verified once by gel electrophoresis and sequencing. Subsequently, primer specificity was controlled by the analysis of the melting curve. Primer efficiency was estimated by serial dilutions of cDNA. Ct values were plotted against the natural logarithm of the template concentration and PCR efficiencies were calculated from the slope of the regression line according to the equation E=10^[-1/slope]^[77]. Beta-2-microglobulin (B2M) was used as an internal reference for normalization in relative quantification [78]. The relative expression ratio (R) of a target gene was calculated using following formula: ratio = (E target ^∆ Ct target (control-treatment)^)/(E ref ^∆ Ct ref (control-treatment)^) [77]. The P(H1) value, calculated with REST Â©2009 (by Corbett Research Pty Ltd/ Qiagen group and M.W. Pfaffl), indicates the probability that the difference between sample and control group is significant [79].

## Competing interests

The authors declare that they have no competing interests.

## Authors’ contributions

ORAC: AK, MJ; viability assay: PG; ROS-assay: AK, KB; ARE-assay: AK, MJ; qPCR: JMG, KB; microarrays: OAW, IPA analysis: MJ, JMG, OAW; experimental design and manuscript conception: MJ, JMG, DF, FÜ; manuscript writing: JMG, MJ; critical revision of the manuscript: DF, FÜ. All authors discussed the results and read and approved the final manuscript.

## Supplementary Material

Additional file 1: Table S1 Top seven networks affected by the treatment of HepG2 cells with Padma 28. **Table S2.** List of primers used for validation of differentially expressed genes by qPCR. Click here for file
