# Supplementary material for: Pathway-focused bioassays and transcriptome analysis contribute to a better activity monitoring of complex herbal remedies
Source: BMC Genomics. 2013 Feb 27;14:133. doi: 10.1186/1471-2164-14-133 (PMC3598787; doi:10.1186/1471-2164-14-133)
Supplement: Additional file 1: Table S1 — Top seven networks affected by the treatment of HepG2 cells with Padma 28. Table S2. List of primers used for validation of differentially expressed genes by qPCR. [file 1471-2164-14-133-S1.pdf]

Additional files

Table S1 – Top seven networks affected by the treatment of HepG2 cells with Padma 28.

Network	Molecules in Network	Score	Focus Molecules	Top Functions
**1**	AREG, **ASNS**, BACE1, CARD8, CLIP1, CTH, DDX6, ENC1, FILIP1L, FSH, GARS, GCLC, **GCLM**, hCG, IKK (complex), ISG20, Lh, MAP4K4, MTHFD2, MYBL1, NFkB (complex), NOC2L, PHKA2, PMAIP1, PSAT1, RAB31, **RAB27A**, RGS3, Sapk, SGK1, STC2, TNFSF4, TRIB3, TXNRD1, VAV3	49	29	Drug Metabolism, Amino Acid Metabolism, Small Molecule Biochemistry
**2**	ACVR1B, Ahr-aryl hydrocarbon-Arnt, **AKR1C1**, **AKR1C2**, ALCAM, **CYP1A1**, **CYP24A1**, CYP2S1, CYP4F11, ERK1/2, Glucose Transporter, HBA1, Hemoglobin, **HMOX1**, HNF4α dimer, HNRNPD, IL11, INHBE, NCOA2, NR1H3, Pias, PXR ligand-PXR-Retinoic acid-RXRα, Rxr, SH2B3, SLC2A1, SLC2A2, SLC2A3, SLC35A2, SLCO1B3, SPP1, T3-TR-RXR, TDGF1, TIA1, unspecific monooxygenase, VitaminD3-VDR-RXR	37	24	Lipid Metabolism, Small Molecule Biochemistry, Vitamin and Mineral Metabolism
**3**	BCOR, C5ORF13, CCNL1, CENPF, Cyclin A, DBNDD1, **DKK1**, ELF4, EMP3, ERBB2, ERK, Estrogen Receptor, EWSR1, GTF2H1, HDAC5, Histone h3, Histone h4, KLF5, NRP1, OSGIN1, OTX1, p85 (pik3r), PDGF BB, PDZK1, PLC gamma, **RBM39**, RBPMS, RNA polymerase II, RUNX1, SERF2, **SLC26A3**, SSH1, UBA2, Ubiquitin, Vegf	36	24	Gene Expression, Cancer, Connective Tissue Disorders
**4**	Actin, ADA, AGPAT9, BLVRB, Caspase, Ck2, CP, CXADR, EIF4EBP1, F Actin, HAL, HNRNPC, Hsp90, IDH3A, IKBKG, IL1, Immunoglobulin, KCNC4, MAP1B, Mapk, MARS, OGG1, PDE4D, Pkc(s), PLAA, Pld, PLD1, PP2A, RGS2, S100A11, SLC6A9, SOS1, SPTAN1, VIL1, WARS	36	24	Molecular Transport, Amino Acid Metabolism, Genetic Disorder
**5**	Alpha Actinin, C5AR1, CD36, CD47, COL2A1, Collagen type I, Collagen type IV, Collagen(s), CTGF, CYR61, Elastase, EPB41L1, FN1, Focal adhesion kinase, Integrin, Integrin alpha 3 beta 1, Integrin alpha 5 beta 1, LRP, LYN, MEP1A, Metalloprotease, Mmp, NQO1, **NUPR1**, PALLD, Pdgf, PI3K, PLAUR, PLG, PVRL2, TF, THBS1, TNS1, TSKU, VCAN	29	21	Cell-To-Cell Signaling and Interaction, Tissue Development, Cell Morphology
**6**	ABCC3, **ABCC4**, ALDH1L1, ALDH8A1, BMP2K (includes EG:55589), C16ORF61, CAR ligand-CAR-Retinoic acid-RXRα, CLYBL, CUTC, FOXJ3, GABRE, GCKR, GCNT3, GIPC2, GPX2, **GSTA1**, GSTCD, HAL, HGD, HNF1A, HNF4A, KIF1B, LSG1, MGST1, MON1B, MRPL15, MRPS12, NOP16, PAFAH2, RUFY3, SENP1, SLC17A2, SUPT3H (includes EG:8464), SUPV3L1, UNC119	29	20	Drug Metabolism, Vitamin and Mineral Metabolism, Molecular Transport
**7**	**AKR1B10**, ARG1, B4GALNT1, C5AR1, CCNA1, **CD14**, CD276, CHD2, CRLF1, CSHL1, Cytochrome p450, FAH, G protein, GPR126, HEG1, HGF, IDE, IFNG, **KLHDC9**, KRR1, LTB4R, MAML2, MIR27B (includes EG:407019), NF2, NOTCH1, OAS3 (includes EG:4940), OGG1, PHF15, PHGDH, **PMAIP1**, **SCHIP1**, SEPP1, TGFB1I1, TNF, YARS	23	17	Cardiovascular System Development and Function, Cell-To-Cell Signaling and Interaction, Inflammatory Response

Gene names in red and green are those identified by microarray analysis as more than two-fold up- or down-regulated, respectively. Genes in bold are modulated more than four-fold.

Table S2 – List of primers used for validation of differentially expressed genes by qPCR.

Gene Symbol	Sequence (5'→3')	Genomic position	Amplicon length
AKR1B10	TCACCCATACCTCACACAGG<br>GGAAACGGATCAGAACCTGG	6th exon<br>7th/8th exon	198 bp
AKR1C1-C4	TACAATAATGAGGAGCAGGTTG<br>CCAAAGCTTTGAAGTGTAGAATA	2nd exon<br>2nd/3rd exon	96 bp
ALDH1L1	CTGATGTGGTGGCAAAATACC<br>TGAATGAGGGTCCAGTTGATG	1st exon<br>1st/2nd exon	175 bp
ASNSß9	TTTATCAGGGGGCTTGGACT<br>CCAGTAAATCGGGGCTGTCT	6th/7th exon<br>7th exon	125 bp
B2M	CTCCGTGGCCTTAGCTGTG<br>TTTGGAGTACGCTGGATAGCCT	1st exon<br>1st/2nd exon	69 bp
CARS	ACGCCAGGTCCTACATCT<br>TTCTCCCGATACTGCTCGAA	3rd/4th exon<br>5th exon	154 bp
CYP1A1	CAGATCAACCATGACCAGAAGC<br>TTCTCACTTAACACCTTGTCGATAGC	6th/7th exon<br>7th exon	101 bp
CYP24A1	GTGGAAACGACAGCAAACAG<br>CCGTGGCACCTGATTCTC	7th/8th exon<br>8th exon	129 bp
CYP2S1	CAGGGCACGGAGGTCTTC<br>ACACGCTTCCCTAAGGAGAA	7th/8th exon<br>8th/9th exon	155 bp
DKK1	ATCACACCAAAGGACAAGAA<br>GGCAAGACAGACCTTCTCCA	3rd/4th exon<br>4th exon	201 bp
FECH	AGCCAAGGAGTGTGGAGTT<br>CTCGTTTGACTGGATGTGTGA	9th/10th exon<br>11th exon	112 bp
GCLC	TGGGAAGGAAGGTGTGTTTC<br>TCAGGATGGTTTGCGATAAA	14th/15th exon<br>16th exon	183 bp
GPX2	AGAATGTGGCTTCGCTCTGA<br>ACAGTTCTCCTGATGTCCA	1st exon<br>1st/2nd exon	131 bp
GSTA1-A3, A5	GAGCCACGGACAAGACTACC<br>GGCCTTCAGCAGAGGGAA	8th exon<br>8th/9th exon	127 bp
HMOX1	AGCAACAAAGTGCAAGATTCTG<br>GGCATAAAGCCCTACAGCAA	4th/5th exon<br>5th exon	141 bp
LGR5	CTCAAGATGAACGTGACCTT<br>TGCTATGGTCCACACTCCAA	16th/17th<br>18th	158 bp
RBM39	TGCCATCCGAGGAAAGATTG<br>TATCAATAGGTTCTCTCACAGG	5th exon<br>6th/7th exon	113 bp
SCN1A	ATACCTCGACCAGGAAACAA<br>CAGATTGATGCGTGACAAAATG	24th/25th<br>25th	168 bp
SUCLG1	ATTGGTGGTAATGCAGAAGAG<br>ATTCCTTGTAGATCGTGGTTC	7th<br>8th/9th	247 bp
WARS	ATCAGGAAGGATTACACCAGC<br>CTCTTTCACTATCTCATCCGTG	9th/10th exon<br>10th exon	132 bp
